# Supplementary material for: Gaidropsarus gallaeciae (Gadiformes: Gaidropsaridae), a New Northeast Atlantic Rockling Fish, with Commentary on the Taxonomy of the Genus
Source: Biology (Basel). 2022 Jun 3;11(6):860. doi: 10.3390/biology11060860 (PMC9219912; doi:10.3390/biology11060860)
Supplement: Supplementary file 1 [file biology-11-00860-s001.zip › biology-1747085-supplementary.pdf]

Table S1. Morphological data of the *Gaidropsarus* species examined as comparative material. TL and SL values are given in mm. The name of each species and the codes of its specimens deposited in the MHNUSC are given in the heading

G. vulgaris	**25196-1**	**25196-2**	**25196-3**	**25196-4**	**25196-5**
TL	320	239	295	262	258
SL	280	210	264	231	225
As % SL					
Head length	23.6	25.1	25.8	24.8	25.9
1st Predorsal length	22.1	23.8	22.7	23.8	24
3rd Predorsal length	36.4	38.1	37.9	36.4	37.8
2nd dorsal base fin length	13.9	13.3	12.5	11.3	12
3rd dorsal base fin length	61.1	57.1	54.9	58.9	57.8
Anal base fin length	43.6	40.5	43.2	42	45.3
Pectoral fin length	15	15.4	15.2	15.1	14.1
Pelvic fin length	19.3	20	17.4	18.4	20.3
Preanal length	51.1	54.8	48.9	52.8	49.3
Body depth	20.4	15.5	16.1	14.8	14.9
Prepectoral length	25.4	24.3	23.5	24.7	24
Prepelvic length	20.7	19	20.1	18.6	20
Caudal peduncle height	7.1	7.6	7.2	7.3	7
As % HL					
Snout length	21.2	24.7	25.8	26.6	24.3
Eye diameter	13.6	12.1	10.5	11.2	11.3
Postorbital length	65.2	55.3	63.7	62.2	64.4
Interorbital space	18.2	16.5	17.7	19.5	19.7
Upper jaw length		44.8	49.3	42.3	44.5
Lower jaw length		37.6	39	40	36.8
Chin barbel length	24.2	20.5	23.6	19.6	20.5
1st dorsal fin ray length	11.4	12.9	11.6	9.5	15.2
Meristic					
3rd dorsal fin rays	60	58	57	59	56
Anal fin rays	50	47	47	50	46
Pectoral fin rays	21	21	20	21	22
Ventral fin rays	8	7	8	7	7
Gill rakers (inner)	1+8	1+9	1+8	1+8	1+8
G. macrophthalmus	**25199-1**	**25199-2**	**25199-3**	**25199-4**	**25199-5**
TL	201	171	157	185	187
SL	180	150	140	166	168
As % SL					
Head length	22.6	23.2	23.1	22.4	22.1
1st Predorsal length	20.6	21.3	22.9	20.5	22.6
3rd Predorsal length	38.3	33.3	33.6	33.1	33.9
2nd dorsal base fin length	11.7	10.7	8.6	10.2	10.1
3rd dorsal base fin length	55.6	63.3	61.4	61.4	59.5
Anal base fin length	46.7	50	49.3	50	45.8

Pectoral fin length	14.8	15.3	15.5	14.9	14.7
Pelvic fin length	15.1	16.1	15.3	9.6	14
Preanal length	46.7	45.3	45.7	44	47.6
Body depth	15.3	14.2	19.5	15	14.9
Prepectoral length	22.2	22	22.9	19.3	25
Prepelvic length	18.9	19.3	18.6	16.3	20.2
Caudal peduncle height	5.3	6	7.1	8.2	9.7
As % HL					
Snout length	21.9	23.2	26	21	23.4
Eye diameter	16	17.7	19.8	20.5	18
Postorbital length	55.5	59.1	54.3	58.4	58.6
Interorbital space	17.9	17.5	20	23	19.7
Upper jaw length	46.4	51.7	52.1	46.9	52.9
Lower jaw length	37.1	43.8	42.6	36.6	44
Chin barbel length	18.8	22.2	23.6	14	22.1
1st dorsal fin ray length	13.8	–	17	14.5	13.7
Meristic					
3rd dorsal fin rays	52	54	54	55	53
Anal fin rays	47	45	47	46	46
Pectoral fin rays	20	22	21	20	19
Ventral fin rays	6	6	6	6	6
Gill rakers (inner)	0+9	1+7	1+8	1+7	1+8
G. ensis	**25197-1**	**25197-2**	**25197-3**	**25197-4**	
TL	230	222	355	245	
SL	198	188	312	213	
As % SL					
Head length	19.7	22	19.6	19.9	
1st Predorsal length	18.7	20.2	19.6	18.8	
3rd Predorsal length	32.3	31.4	32.1	29.1	
2nd dorsal base fin length	11.1	8	10.9	11.3	
3rd dorsal base fin length	62.1	64.4	59.3	62.4	
Anal base fin length	43.9	46.3	41.7	39.9	
Pectoral fin length	17	19.8	17.9	18.5	
Pelvic fin length	17	21.7	20.7	19.1	
Preanal length	48	48.9	50	49.3	
Body depth	16.7	25.2	18.2	18.4	
Prepectoral length	20.2	21.3	17.9	17.8	
Prepelvic length	14.6	16	15.4	12.7	
Caudal peduncle height	6.9	7.2	5.5	6.7	
As % HL					
Snout length	23.6	23.7	27.9	24.3	
Eye diameter	20	17.3	18	21.1	
Postorbital length	56.2	59.1	54.1	54.7	
Interorbital space	21.8	25.1	14.4	20.8	
Upper jaw length	51.3	64.8	53	45.3	
Lower jaw length	42.7	60.3	40.1	36.1	

Chin barbel length	15.1	17.1	16.6	20.8
1st dorsal fin ray length	110	82.1	141.5	97.2
Meristic				
3rd dorsal fin rays	59	55	56	56
Anal fin rays	46	46	43	44
Pectoral fin rays	22	22	23	22
Ventral fin rays	7	7	7	7
Gill rakers (inner)	1+10	1+9	1+13	1+11
G. argentatus	**25198-1**	**25198-2**	**25198-3**	
TL	330	282	305	
SL	290	246	266	
As % SL				
Head length	23.6	25.1	24.1	
1st Predorsal length	20.7	21.5	22.6	
3rd Predorsal length	31.7	34.6	36.8	
2nd dorsal base fin	10.3	11.4	8.6	
3rd dorsal base fin	62.4	58.9	57.1	
Anal base fin length	39.3	39.8	38.1	
Pectoral fin length	16.1	16.8	18.9	
Pelvic fin length	18.1	20.7	21.5	
Preanal length	51.4	52.8	53.4	
Body depth	23.5	18.9	15.6	
Prepectoral length	20.7	28	24.1	
Prepelvic length	17.2	19.9	16.9	
Caudal peduncle	6.6	7.4	5.5	
As % HL				
Snout length	26	25.2	27	
Eye diameter	16.7	16.4	14.8	
Postorbital length	57.3	18.4	58.1	
Interorbital space	17.8	19.4	22.2	
Upper jaw length	46.3	44.7	47.7	
Lower jaw length	41.1	36.6	38.3	
Chin barbel length	19.8	23.8	22.8	
1st dorsal fin ray length	40.6	30.3	35.6	
Meristic				
3rd dorsal fin rays	57	57	55	
Anal fin rays	50	43	43	
Pectoral fin rays	25	24	23	
Ventral fin rays	7	7	7	
Gill rakers (inner)	1+9	1+10	1+9	
G. mediterraneus	**25195-1**	**25195-2**		
TL	277	190		
SL	220	169		
As % SL				
Head length	20.8	24		
1st Predorsal length	18.6	18.9		

3rd Predorsal length	32.3	38.5
2nd dorsal base fin	13.2	17.8
3rd dorsal base fin	54.1	55
Anal base fin length	45	45
Pectoral fin length	12.3	14.6
Pelvic fin length	13	15.5
Preanal length	44.1	48.5
Body depth	14	19.3
Prepectoral length	20.5	22.5
Prepelvic length	15	17.2
Caudal peduncle	5.5	6.3
As % HL		
Snout length	21.4	23.4
Eye diameter	15.2	13.8
Postorbital length	63.5	62.8
Interorbital space	17.9	19
Upper jaw length	45.6	42.9
Lower jaw length	40.2	38.9
Chin barbel length	15.3	18.5
1st dorsal fin ray length	14.9	–
Meristic		
3rd dorsal fin rays	55	48
Anal fin rays	47	45
Pectoral fin rays	17	15
Ventral fin rays	7	8
Gill rakers (inner)	0+6	1+5
G. mediterraneus	25195-1	25195-2
TL	277	190
SL	220	169
As % SL		
Head length	20.8	24
1st Predorsal length	18.6	18.9
3rd Predorsal length	32.3	38.5
2nd dorsal base fin	13.2	17.8
3rd dorsal base fin	54.1	55
Anal base fin length	45	45
Pectoral fin length	12.3	14.6
Pelvic fin length	13	15.5
Preanal length	44.1	48.5
